# Restricted kinematic alignment leads to uncompromised osseointegration of cementless total knee arthroplasty

**DOI:** 10.1007/s00167-020-06427-1

**Published:** 2021-01-16

**Authors:** Guillaume Laforest, Lazaros Kostretzis, Marc-Olivier Kiss, Pascal-André Vendittoli

**Affiliations:** 1 Surgery Department, Hôpital Maisonneuve-Rosemont, Université de Montréal, 5415 Boul l’Assomption, Montreal, QC H1T 2M4 Canada; 2 Clinique Orthopédique Duval, Laval, QC Canada; 3 Personalized Arthroplasty Society, Montreal, Canada

**Keywords:** Total knee arthroplasty, Total knee replacement, Uncemented, Kinematic alignment, Mechanical, Survival

## Abstract

**Purpose:**

While kinematic alignment (KA) total knee arthroplasty (TKA) with cemented implants has been shown to provide equivalent or better results than mechanical alignment, its combination with cementless fixation has not yet been documented. The purpose of this study is to report (1) revision rate and causes, (2) clinical results based on patient report outcome measures (PROMs), and (3) radiological signs of implant dysfunction in patients with an uncemented TKA implanted with restricted KA (rKA), after a minimum follow-up of 2 years.

**Methods:**

This study included the first 100 consecutive uncemented cruciate retaining TKAs implanted between November 2015 and February 2018 by a single surgeon following rKA principles. At last follow-up, all adverse events and PROMs assessed by WOMAC, KOOS, and FJS scores were documented. Radiographic evaluation was performed to identify signs of implant loosening.

**Results:**

After a mean follow-up of 49 months (32, 60), no implant revision was performed for aseptic loosening. Three revisions were performed: one for malalignment, one for a deep infection, and one for instability. The mean WOMAC score was 20.1 (0–79, 21.3), the mean KOOS score was 71.5 (19.0–96.6, 19.8), and the mean FJS score was 65.9 (0–100, 29.6). No radiological evidence of implant aseptic loosening or osteolysis was identified.

**Conclusion:**

This study shows that in 99% of our cases, rKA combined with the tested cementless TKA implant allowed for adequate secondary fixation and good functional outcomes in the short term. Favourable mid- to long-term implant survivorship is anticipated.

**Level of evidence:**

III.

## Introduction

Total knee arthroplasty (TKA) is the most common joint replacement procedure performed in the world [8]. TKA failure leading to revision is most often the result of aseptic loosening, which occurs at a higher rate in younger patients [9, 29, 33] and in morbidly obese patients [29, 40, 42]. These two groups of patients are thought to subject the implants to greater mechanical stress [3, 23, 40, 44]. To overcome these important challenges, uncemented TKA fixation was proposed to improve long-term fixation [11, 14, 30, 33].

Providing a forgotten TKA remains a challenge, despite important improvements in implant designs, fixation methods, and precision of implantation [8, 16]. This leads to the question whether traditional and systematic mechanical alignment (MA) is the ideal method [17, 36], given that coronal knee alignment varies significantly in both non-arthritic [32] and arthritic populations [2]. A comprehensive description of different lower limb alignment phenotypes has been proposed by Hirschmann et al. [18–20]. Thus, the concept of kinematic alignment (KA) has gained momentum in the last few years because it uses personalized bone resections to recreate the individual pre-arthritic knee anatomy, phenotype, and ligament laxity [2, 35].

KA implies deviating from the systemic neutral lower limb alignment goal of MA and raises the concern that keeping the lower limb in varus will lead to medial compartment overload and tibial component loosening [6]. Furthermore, it is feared that KA non-neutral alignment may jeopardize primary and/or secondary uncemented TKA implant fixation [14]. To our knowledge, all studies reporting KA TKA clinical results included cemented implants, and, therefore, the safety of KA with uncemented implants is still unknown. The purposes of this study are to assess: (1) the revision rate, its causes, and radiological signs of implant dysfunction, (2) the clinical results measured by different patient reported outcome measures (PROMs), and (3) radiographic coronal alignment measurements, after a minimum follow-up of 2 years, in a cohort of 100 patients who underwent a restricted KA (rKA) uncemented TKA. Our hypothesis is that cementless TKA implants can be safely combined with rKA in the short term.

## Methods

### Patients

**Fig. 1:**
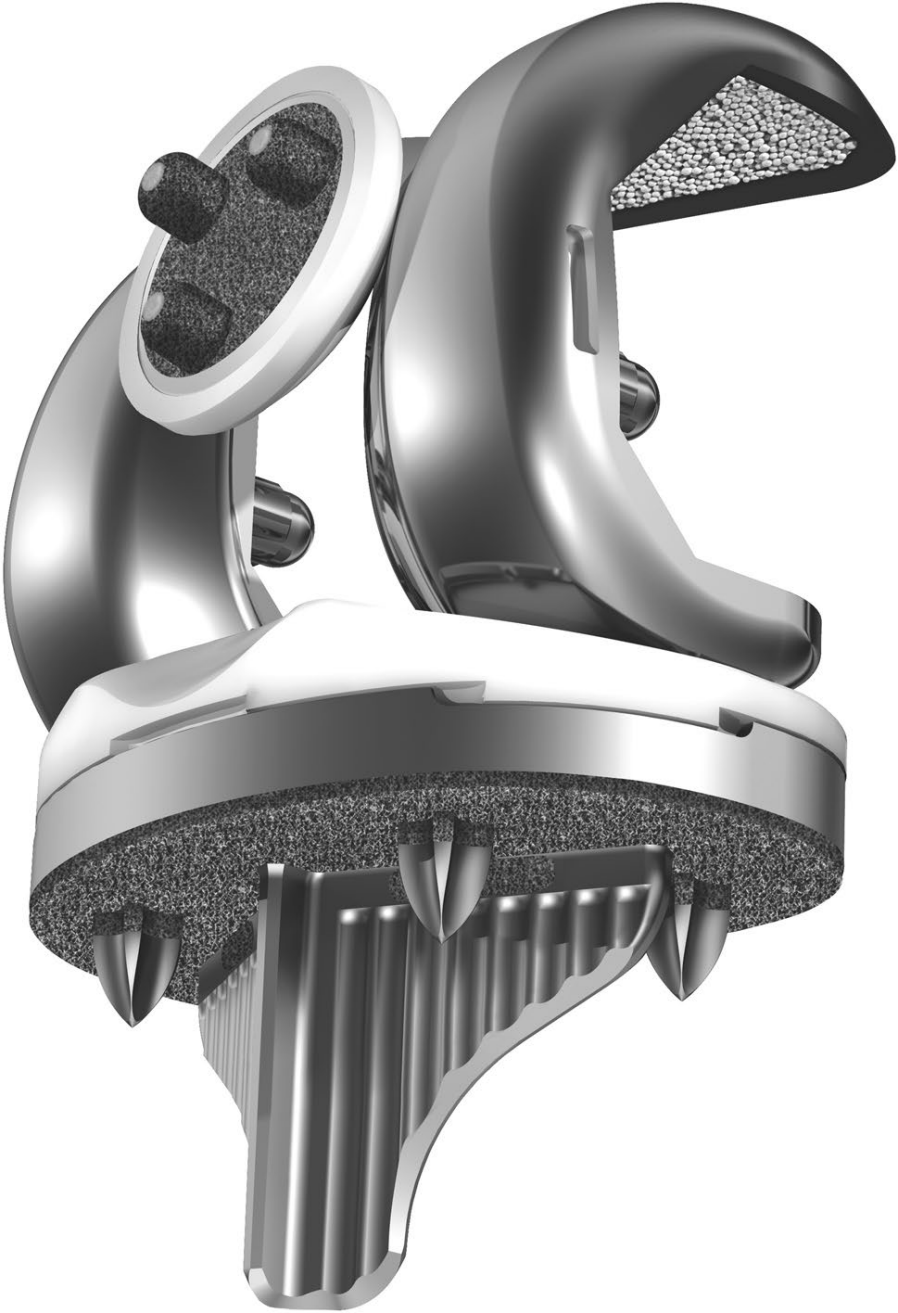
Triathlon™ uncemented cruciate-retaining TKA (Stryker)

The senior author had been performing rKA since 2011 and implanted his first uncemented cruciate-retaining TKAs (Triathlon Tritanium, Stryker, Mahwah, USA, Fig. 1) in November 2015. By February 2018, the surgeon had performed 133 primary TKAs in his academic practice. Among these, 3 cases were pre-operatively considered inappropriate to receive the studied uncemented CR TKA implant (1 tumoural case requiring a cemented stemmed implant and 2 patients with ligamentous incompetence requiring higher implant constraint) and 27 TKAs were included in another study evaluating a different implant. Of the 103 cases remaining, inaccurate intra-operative femoral bone cuts precluded uncemented implant use in 1 patient, and 2 patients refused to participate in the study. Hence, 100 uncemented TKA cases were included in this study. Patients’ demographics are presented in Table 1. To review patients’ charts and obtain patients’ PROMs at last follow-up, ethics and scientific committee approvals were obtained from our institution, and informed consent was obtained from all participants.

**Table 1:** Patients demographics and surgical details

Gender: male/female ratio	24/76
Age (mean, range, SD)	67.4, 44.5–87.5, 10.0
BMI (mean, range, SD)	32.3, 21.8–53.9, 6.3
Diagnosis	
Primary OA	97%
Post-traumatic OA	3%
Surgical time (mean, range, SD)	56.2, 36–101, 12.0
Patella resurfaced %	55

Radiographic measurements	Pre-operative	Post-operative	*p* value
HKA (mean, range, SD)	− 1.4, − 10.5–10.0, 4.1	− 0.9, − 4.4–3.3, 2.0	*p* = 0.203
LDFA (mean, range, SD)	91.8, 86.0–99.0, 2.7	92.3, 87.2–95.4, 1.8	*p* = 0.091
MPTA (mean, range, SD)	86.7, 79.9–85.4, 2.6	86.8, 84.2–0.0, 1.5	*p* = 0.951

*BMI* body mass index, *HKA* arithmetic mechanical hip–knee–ankle angle (LDFA + MPTA), negative values represent a varus alignment, *LDFA* lateral distal femoral angle (mechanical), *MPTA* medial proximal tibial angle (mechanical), *OA* osteoarthritis, *SD* standard deviation from the mean

### Procedure

**Fig. 2:**
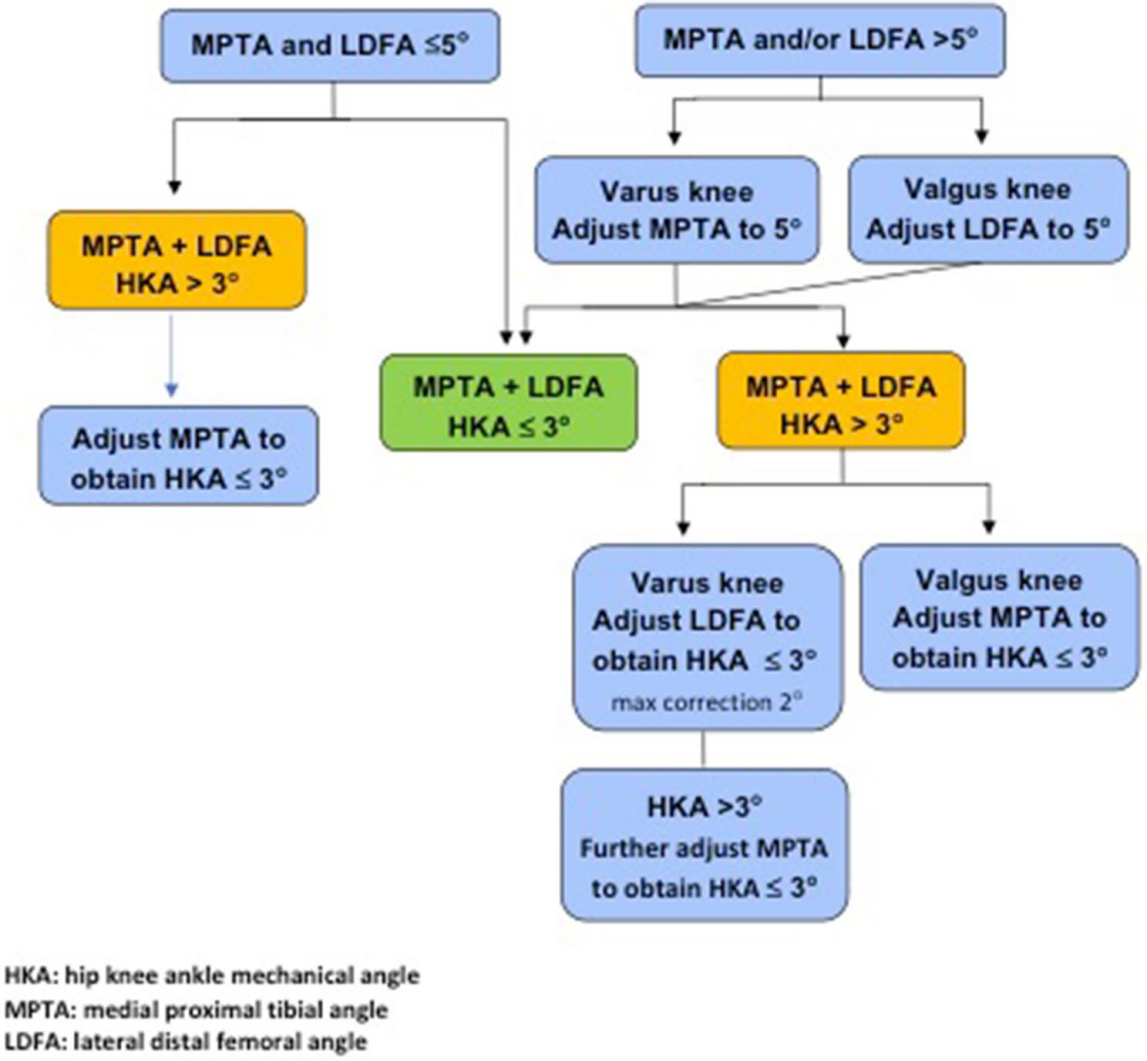
Vendittoli’s restricted kinematic alignment protocol

An anterolateral skin incision and medial mid vastus parapatellar arthrotomy without tourniquet were used for all cases. Vendittoli’s rKA protocol was followed, using optical computer navigation (Orthomap ASM, Stryker, MI, USA) (Table 2). Cartilage and bone loss thicknesses were estimated based on comparison with intact areas, and we aimed to restore the patient’s pre-arthritic alignment. For example, in a varus knee, the distal femoral and proximal tibial cut resections were, respectively, set at 8 and 9 mm (implants’ thicknesses) for unworn cartilage surfaces of the lateral femoral condyle and tibial plateau. Then, cartilage wear thickness was assessed on the medial side bone surfaces (no wear = 0 mm, partial cartilage wear = 1 mm, and subchondral bone exposed = 2 mm) [27]. Cut angle was thus adjusted to reach the desired medial resection thickness (example, for a patient with 2 mm of medial tibial wear, a 7-mm medial resection and a 9-mm lateral resection were used). Resections only differed from patient anatomy when the measured angles fell outside the predefined “safe range” as depicted in Fig. 2. To resurface the posterior condyles, a posterior referencing guide was set to neutral rotation, thus resecting only the implant thicknesses on both posterior condyles (no femoral rotation modification). Tibial component rotation was set by its alignment with the trial femoral component with the knee in extension. In the cases where the resection pieces did not appear to match the computer plan or when ligament laxities assessed with trial implants was outside the expected native ligament laxity range [15], resection accuracy was confirmed by calliper measurements and cut adjustment was performed when needed. The uncemented cruciate-retaining Triathlon (Stryker) prosthesis was implanted in all cases. Its femoral component has a beaded (CrCo) and peri-apatite-coated porous surface, and its tibial component has a porous titanium coating and 4 cruciform pegs for primary fixation (Fig. 1). In selected cases, an uncemented patellar implant backed by porous titanium and with 3 pegs was implanted.

**Table 2:** The five Vendittoli’s restricted kinematic alignment principles

Principle	Description
1	HKA limits ± 3**°** Arithmetic combination of LDFA and MPTA should be ± 3°
2	Joint obliquity limits to 5**°** LDFA and MPTA maximum 85–95**°**
3	Restore native ligament laxities No ligamentous releases should be performed unless anatomical bone adjustments are required by the protocol boundaries. Ligamentous releases are usually required with anatomical corrections > 3 degrees
4	Adjust the most contributing bone to the alignment deviation but favour femoral anatomy preservation (see Fig. 2)
5	Resurface the intact compartment (remove a bone and cartilage thickness equivalent to the implant thickness) and adjust the opposite compartment resection thickness Varus = lateral pivot point Valgus = medial pivot point

*LDFA* lateral distal femoral angle (mechanical), *MPTA* medial proximal tibial angle (mechanical), *HKA* arithmetic mechanical hip–knee–ankle angle (LDFA + MPTA)

### Methods of assessment

All patients were included in our prospective institution database collection system. A retrospective patients’ chart review was performed to seek any adverse events requiring reoperation or revision surgery during the follow-up period. All post-operative radiographs were evaluated following the modern Knee Society Radiographic Evaluation System to assess radiolucent lines, osteolysis, and signs of component loosening [28]. PROMs (WOMAC, KOOS, and Forgotten Joint Scores) were administered in the outpatient clinic at last follow-up by a single research assistant. Radiographic pre- and post-operative coronal orientation measurements were taken using the lateral distal femoral angle (LDFA), the medial proximal tibial angle (MPTA), and the hip–knee–ankle angle (HKA). Using a digitized image and measurement tools, the same evaluator took all measurements as described previously [5, 10, 28, 41].

### Statistical analyses

Sample size calculation using a power of 80%, a *p* value of < 0.05, and 100 cases, showed that the minimal detectable difference in the revision rate between our cohort and the reported result with cemented rKA would be 7% [22]. Continuous data are presented with mean, minimum, maximum, and standard deviation. Since our number of cases is 100, proportions are presented with percentage alone. Comparisons of the pre-operative and post-operative continuous data were analysed using a paired Student’s *T* tests. A significance level of *p* = 0.05 (two-sided) was used for all tests. The analyses were performed using the SPSS software version 26 (SPSS Inc., Chicago, IL, USA).

## Results

**Fig. 3:**
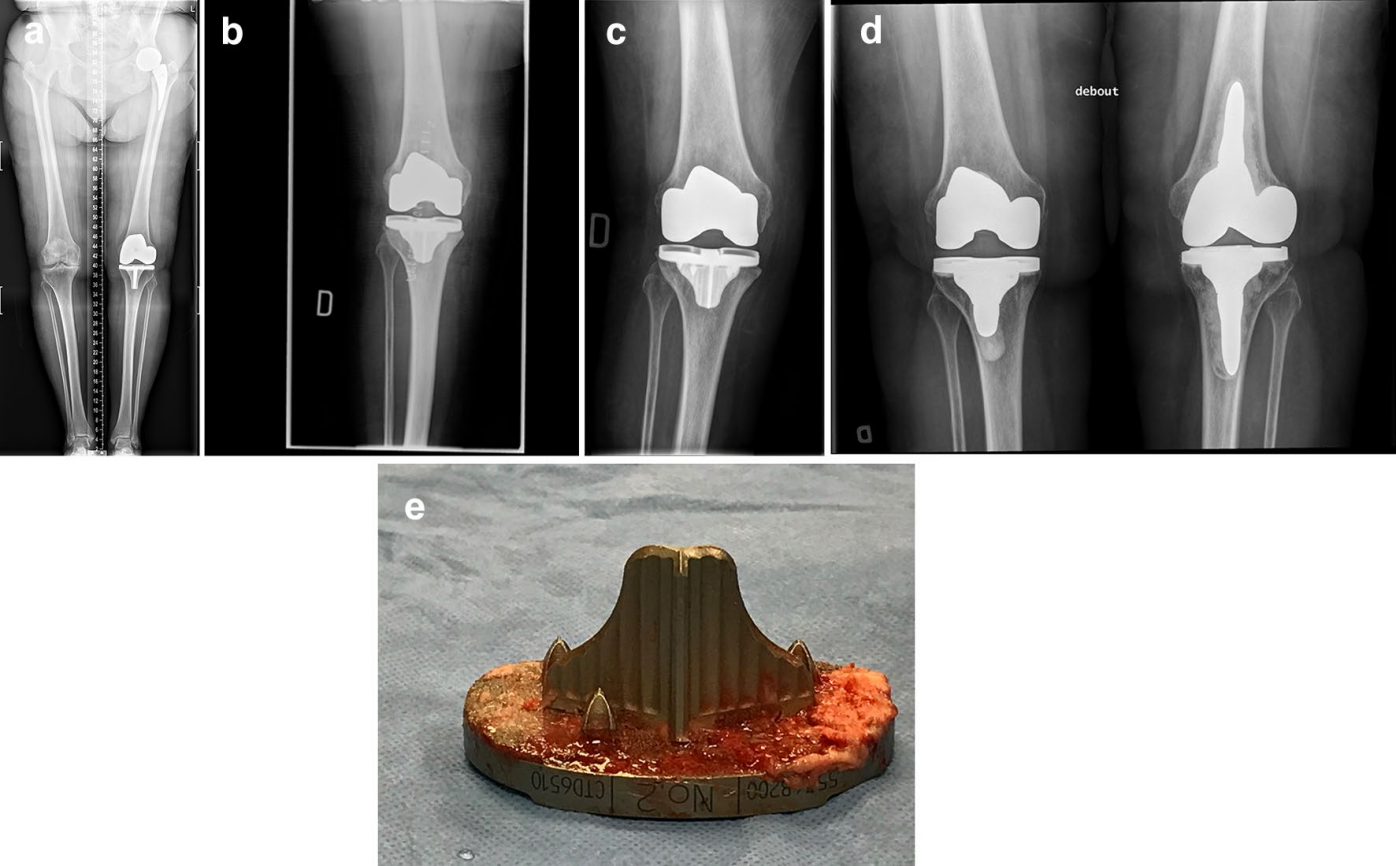
**a** Pre-op long leg AP view radiograph of a 76-year-old female with severe medial OA where LDFA is measured at 87.5° (valgus) and MPTA at 88.1° (varus), leading to an arithmetic HKA of 0.6°. **b** Immediate post-op AP radiograph showing uncemented TKA implants in acceptable orientation: 88.0° LDFA, 0° MPTA and arithmetic HKA of 2.0°. **c** Patient sustained a fall in stairs 4 weeks after surgery. Sudden and persisting pain and swelling were present. This is an AP view radiograph, 8 weeks post-op, showing a 5° valgus shift of the tibial implant (MPTA changed from 90° to 95° (LDFA was maintained at 88.0°). **d** Patient being unsatisfied with her lower limb alignment (HKA: 7° valgus) and having medial knee pain (medial collateral ligament over tensioned), she requested a TKA revision surgery. During revision procedure, the femoral implant was well fixed and considered well aligned. Tibial implant was revised alone, changing its orientation and using a cemented version. AP view radiograph post revision showing tibial implant’s MPTA at 88.0°, combined leading to an arithmetic HKA of 0° when combined with the femoral implant LDFA of 88.0°. **e** Removal of the well-fixed uncemented tibial implant was demanding, especially to break the osseous bonding behind the keel. Here is a photograph of the removed uncemented tibial implant where cancellous bone attachment is observed on the whole porous surface

After a mean post-operative time of 49 months (32–60), 1 patient (2 knees) was deceased from causes unrelated to his TKA and no patient was lost to follow-up. Three TKAs (3%) were revised. One patient, a 76-year-old female fell on stairs 4 weeks after surgery and had a 5° tibial implant valgus shift (Fig. 3). The patient’s persistent discomfort with the malalignment led to revision surgery 13 months after primary implantation. During revision, the tibial implant was well fixed in the valgus malposition and was revised with a cemented implant. One 62-year-old male patient underwent two-stage revision for a deep and chronic infection 21 months after initial surgery (micro-organism was *Cutibacterium acnes*). Thirty-three months after revision, the patient is free from infection. The final revision was performed on a 78-year-old male patient 9 months after initial surgery for persisting pain and swelling secondary to flexion instability linked to femoral and tibial implants under sizing. During revision, implants were solidly integrated. Increasing the femoral implant size from 3 to 5 with posterior augments and increasing the tibial implant size from 4 to 5 solved his instability symptoms. At last follow-up, radiographic analyses did not reveal evidence of implant loosening, osteolysis, radiolucency, or reactive changes.

At last follow-up, the mean WOMAC score was 20.1 (0–79, 21.3), the mean KOOS score was 71.5 (19.0–96.6, 19.8), and the mean FJS score was 65.9 (0–100, 29.6).

Pre- and post-operative radiographic measurements are provided in Table 1 and fall within expected values according to the rKA protocol.

## Discussion

The most important finding of the present study was that cementless TKA implants can be safely combined with rKA in the short-term, thus supporting our hypothesis. To the best of our knowledge, this is the first study to report results of cementless rKA TKA. In our study, we showed that primary fixation of the implant was sufficient in 99% of cases.

Improving patient satisfaction, function, and survivorship following TKA remains a subject at the forefront of orthopaedic research. This study aimed to report clinical and radiological outcomes of patients who underwent uncemented rKA TKA. Regarding secondary fixation, clinical evaluation and radiographic review after a mean follow-up of 34 months did not show lack of integration or signs of loosening. Even the patient with early mobilisation and malalignment of the tibial component, the implant was well-fixed (osteointegrated) at time of revision.

The optimal implant orientation when performing a TKA remains a pertinent and unanswered question [17]. KA aims to restore the individual’s pre-arthritic or native limb and joint line alignment by resurfacing the knee joint with the intent of reproducing a more natural joint feeling. There is a rapidly growing amount of evidence supporting the safety of this technique. Meta-analyses of available comparative studies have demonstrated either equivalent or favourable early clinical results for cemented TKA KA over MA [35]. Laende et al., using radiostereometric analysis to predict long-term implant survivorship in a randomized controlled trial (RCT) comparing MA to KA cemented TKA, found no difference in implant migration after 2 years [25]. Howell et al. reported a revision rate of 1.5% in 220 cemented KA TKAs at 10 years post-operatively [21]. A combined Australian and New Zealand registry study including 20,512 cases of cemented Triathlon cruciate-retaining TKA reported similar revision rates at 7 years for KA and other alignment techniques [24]. Our study results fall in line with the available literature for MA, which supports rKA as a safe and reliable technique. One case in our study required revision for flexion instability possibly due to undersizing of the femoral and tibial implants. Although review of the surgical protocol did not reveal an obvious reason for this problem, inadvertent anterior translation setting of the posterior referencing femoral sizing guide is suspected.

Deviating from neutral alignment with KA is still a concern for many surgeons [1]. One may suggest that some knee anatomies may be inherently biomechanically inferior and could jeopardize uncemented TKA primary and/or secondary fixation. With persisting uncertainties about joint load and its alignment deviation from neutral, KA combined with cementless implant needed to be tested, particularly because cementless TKA implant fixation posed a challenge to the scientific community for many years [4, 29, 31, 33]. First-generation cementless designs had poor results associated with early loosening; however, contemporary evidence shows survivorship similar to that of cemented implants [31, 43, 44]. The main success factor for this is satisfactory primary fixation with minimal micromotion allowing bone integration to the porous surface [7, 14]. The current study’s revision rate was 3%, and no case revealed aseptic loosening or a lack of secondary integration, thereby once again supporting our hypothesis. Cementless primary fixation as a potential cause of failure was suspected in only one case of implant mobilization after a significant fall in a patient with no known history of osteoporosis but with experience of a periprosthetic fracture following a previous total hip arthroplasty. It is unsure if a cemented fixation would have prevented this issue. In a similar study, following the same rKA principles and implantation technique but with the cemented Triathlon implants, no revisions were required among 100 consecutive and unselected TKAs after a mean follow-up of 2.4 years [22]. In a RCT comparing cemented and uncemented MA Triathlon in 141 patients, Nam et al. found no radiographic evidence of component subsidence or loosening in either cohort at an average of 2 years post-operatively [33]. They reported one revision for deep infection in the cemented group and no revision in the cementless group.

PROMs obtained in our study are similar to those available in the literature for both cemented and cementless MA TKAs or cemented KA TKAs [44]. In the same previously mentioned RCT, Nam et al. found similar PROMs after an average of 2 years for cemented and cementless MA Triathlon TKAs [33]. Miller et al. also demonstrated similar improvements in functional scores between cemented and cementless TKA cohorts [29]. Two systematic reviews of short-term cemented TKAs results found similar or better functional results with KA over MA [13, 26]. This suggests that rKA combined with cementless fixation TKA should provide comparable or better PROMs results than mechanical alignment with cemented or uncemented fixation.

Regarding radiographic analyses, as expected, there was a significant change in standing coronal alignment between pre-operative mean HKA and post-operative mean HKA from 4.9° to 1.4° varus after surgery. The MPTA, LDFA, and HKA mean values, variation, and range reflect the restoration of individual pre-arthritic alignment with rKA boundaries. On the other hand, with recent understanding that lower limb alignment may vary significantly during gait cycle, it remains unclear as to what influence bipodal static alignment has on TKA articular load [12]. Despite not aiming at neutral limb alignment, KA, in gait analyses, produced a lower knee adduction moment and medial tibial compartment load and more normal gait than MA TKA [34]. Studies have found that intra-operative forces in the medial and lateral compartments of patients with outlier alignment were comparable with those with in-range alignment [37–39]. In our study, radiographic evaluations conducted at last follow-up did not show signs of a lack of fixation.

Our study has limitations. First, the relatively short-term follow-up is an obvious drawback. However, given that uncemented fixation is the most at risk of failure in the first year, we believe our study’s length was sufficient to capture all instances of short-term failure [4, 7]. Once implant osseointegration is achieved, the risk of implant loosening drops drastically, and therefore, we believe the potential for long-term success is great [14]. Second, this consecutive clinical case series is based on a limited number of patients from only one surgeon using a specific rKA protocol, and thus, the results may not be generalizable to all patient populations. Nevertheless, we believe our selection bias was limited. The patients included in our study were unselected and were subjected to very few exclusion criteria. In particular, age, body mass index (BMI), pre-operative alignment, or joint degeneration severity were not part of the exclusion criteria. Therefore, our patients are expected to correspond to the full spectrum of patients that one would expect to see in a public clinical practice. Finally, collecting data that are recorded in standard practice minimized information bias.

We believe that this study can serve as a stepping stone for other surgeons to combine uncemented fixation and rKA, two methods that have already shown much promise independently and are rapidly gaining popularity.

## Conclusion

This study showed that the most crucial period for osseointegration of uncemented TKA was not negatively impacted by our rKA protocol and that favourable mid- to long-term implant survivorship is anticipated. Since this is the first study of its kind, it should be used to promote the adoption of this method and eventually build up the supporting evidence for its continued use.

## Abbreviations

BMI: Body mass index
CrCo: Cobalt-chrome
FJS: Forgotten Joint Score
HKA: Hip–knee–ankle angle
KA: Kinematic alignment
KOOS: Knee Injury and Osteoarthritis Outcome Score
LDFA: Lateral distal femoral angle
MA: Mechanical alignment
MPTA: Medial proximal tibial angle
PROMs: Patient reported
RCT: Randomized controlled trial
rKA: Restricted kinematic alignment
TKA: Total knee arthroplasty
WOMAC: Western Ontario and McMaster Universities Osteoarthritis Index

## References

[1] Abdel MP, Oussedik S, Parratte S, Lustig S, Haddad FS (2014). Coronal alignment in total knee replacement: historical review, contemporary analysis, and future direction. Bone Joint J.

[2] Almaawi AM, Hutt JRB, Masse V, Lavigne M, Vendittoli PA (2017). The impact of mechanical and restricted kinematic alignment on knee anatomy in total knee arthroplasty. J Arthroplasty.

[3] Bagsby DT, Issa K, Smith LS, Elmallah RK, Mast LE, Harwin SF (2016). Cemented vs cementless total knee arthroplasty in morbidly obese patients. J Arthroplasty.

[4] Behery OA, Kearns SM, Rabinowitz JM, Levine BR (2017). Cementless vs cemented tibial fixation in primary total knee arthroplasty. J Arthroplasty.

[5] Bellemans J, Colyn W, Vandenneucker H, Victor J (2012). The Chitranjan Ranawat award: is neutral mechanical alignment normal for all patients? The concept of constitutional varus. Clin Orthop Relat Res.

[6] Blakeney WG, Vendittoli P-A, Rivière C, Vendittoli P-A (2020). Restricted kinematic alignment: the ideal compromise?. Personalized hip and knee joint replacement.

[7] Bouras T, Bitas V, Fennema P, Korovessis P (2017). Good long-term results following cementless TKA with a titanium plasma coating. Knee Surg Sports Traumatol Arthrosc.

[8] Bryan S, Goldsmith LJ, Davis JC, Hejazi S, MacDonald V, McAllister P (2018). Revisiting patient satisfaction following total knee arthroplasty: a longitudinal observational study. BMC Musculoskelet Disord.

[9] Carr AJ, Robertsson O, Graves S, Price AJ, Arden NK, Judge A (2012). Knee replacement. Lancet.

[10] Cherian JJ, Kapadia BH, Banerjee S, Jauregui JJ, Issa K, Mont MA (2014). Mechanical, anatomical, and kinematic axis in TKA: concepts and practical applications. Curr Rev Musculoskelet Med.

[11] Chong DY, Hansen UN, van der Venne R, Verdonschot N, Amis AA (2011). The influence of tibial component fixation techniques on resorption of supporting bone stock after total knee replacement. J Biomech.

[12] Clement J, Blakeney W, Hagemeister N, Desmeules F, Mezghani N, Lowry V (2019). Hip-Knee-Ankle (HKA) angle modification during gait in healthy subjects. Gait Posture.

[13] Courtney PM, Lee GC (2017). Early outcomes of kinematic alignment in primary total knee arthroplasty: a meta-analysis of the literature. J Arthroplasty.

[14] Dalury DF (2016). Cementless total knee arthroplasty: current concepts review. Bone Joint J.

[15] Deep K (2014). Collateral ligament laxity in knees: what is normal?. Clin Orthop Relat Res.

[16] Eichler D, Beaulieu Y, Barry J, Masse V, Vendittoli PA (2020). Perception of a natural joint after total knee arthroplasty. J Arthroplasty.

[17] Hirschmann MT, Becker R, Tandogan R, Vendittoli PA, Howell S (2019). Alignment in TKA: what has been clear is not anymore!. Knee Surg Sports Traumatol Arthrosc.

[18] Hirschmann MT, Hess S, Behrend H, Amsler F, Leclercq V, Moser LB (2019). Phenotyping of hip-knee-ankle angle in young non-osteoarthritic knees provides better understanding of native alignment variability. Knee Surg Sports Traumatol Arthrosc.

[19] Hirschmann MT, Moser LB, Amsler F, Behrend H, Leclercq V, Hess S (2019). Phenotyping the knee in young non-osteoarthritic knees shows a wide distribution of femoral and tibial coronal alignment. Knee Surg Sports Traumatol Arthrosc.

[20] Hirschmann MT, Moser LB, Amsler F, Behrend H, Leclerq V, Hess S (2019). Functional knee phenotypes: a novel classification for phenotyping the coronal lower limb alignment based on the native alignment in young non-osteoarthritic patients. Knee Surg Sports Traumatol Arthrosc.

[21] Howell SM, Shelton TJ, Hull ML (2018). Implant survival and function ten years after kinematically aligned total knee arthroplasty. J Arthroplasty.

[22] Hutt JR, LeBlanc MA, Masse V, Lavigne M, Vendittoli PA (2016). Kinematic TKA using navigation: surgical technique and initial results. Orthop Traumatol Surg Res.

[23] Julin J, Jamsen E, Puolakka T, Konttinen YT, Moilanen T (2010). Younger age increases the risk of early prosthesis failure following primary total knee replacement for osteoarthritis. A follow-up study of 32,019 total knee replacements in the Finnish Arthroplasty Register. Acta Orthop.

[24] Klasan A, de Steiger R, Holland S, Hatton A, Vertullo CJ, Young SW (2020). Similar risk of revision after kinematically aligned, patient-specific instrumented total knee arthroplasty, and all other total knee arthroplasty: combined results from the Australian and New Zealand Joint Replacement Registries. J Arthroplasty.

[25] Laende EK, Richardson CG, Dunbar MJ (2019). A randomized controlled trial of tibial component migration with kinematic alignment using patient-specific instrumentation versus mechanical alignment using computer-assisted surgery in total knee arthroplasty. Bone Joint J.

[26] Lee YS, Howell SM, Won YY, Lee OS, Lee SH, Vahedi H (2017). Kinematic alignment is a possible alternative to mechanical alignment in total knee arthroplasty. Knee Surg Sports Traumatol Arthrosc.

[27] Li G, Park SE, DeFrate LE, Schutzer ME, Ji L, Gill TJ (2005). The cartilage thickness distribution in the tibiofemoral joint and its correlation with cartilage-to-cartilage contact. Clin Biomech.

[28] Meneghini RM, Mont MA, Backstein DB, Bourne RB, Dennis DA, Scuderi GR (2015). Development of a modern knee society radiographic evaluation system and methodology for total knee arthroplasty. J Arthroplasty.

[29] Miller AJ, Stimac JD, Smith LS, Feher AW, Yakkanti MR, Malkani AL (2018). Results of cemented vs cementless primary total knee arthroplasty using the same implant design. J Arthroplasty.

[30] Mont MA, Gwam C, Newman JM, Chughtai M, Khlopas A, Ramkumar PN (2017). Outcomes of a newer-generation cementless total knee arthroplasty design in patients less than 50 years of age. Ann Transl Med.

[31] Mont MA, Pivec R, Issa K, Kapadia BH, Maheshwari A, Harwin SF (2014). Long-term implant survivorship of cementless total knee arthroplasty: a systematic review of the literature and meta-analysis. J Knee Surg.

[32] Moser LB, Hess S, Amsler F, Behrend H, Hirschmann MT (2019). Native non-osteoarthritic knees have a highly variable coronal alignment: a systematic review. Knee Surg Sports Traumatol Arthrosc.

[33] Nam D, Lawrie CM, Salih R, Nahhas CR, Barrack RL, Nunley RM (2019). Cemented versus cementless total knee arthroplasty of the same modern design: a prospective, randomized trial. J Bone Joint Surg Am.

[34] Niki Y, Nagura T, Nagai K, Kobayashi S, Harato K (2018). Kinematically aligned total knee arthroplasty reduces knee adduction moment more than mechanically aligned total knee arthroplasty. Knee Surg Sports Traumatol Arthrosc.

[35] Nisar S, Palan J, Rivière C, Emerton M, Pandit H (2020). Kinematic alignment in total knee arthroplasty. EFORT Open Rev.

[36] Riviere C, Vigdorchik JM, Vendittoli PA (2019). Mechanical alignment: the end of an era!. Orthop Traumatol Surg Res.

[37] Roth JD, Howell SM, Hull ML (2018). Kinematically aligned total knee arthroplasty limits high tibial forces, differences in tibial forces between compartments, and abnormal tibial contact kinematics during passive flexion. Knee Surg Sports Traumatol Arthrosc.

[38] Shelton TJ, Howell SM, Hull ML (2019). Is there a force target that predicts early patient-reported outcomes after kinematically aligned TKA?. Clin Orthop Relat Res.

[39] Shelton TJ, Nedopil AJ, Howell SM, Hull ML (2017). Do varus or valgus outliers have higher forces in the medial or lateral compartments than those which are in-range after a kinematically aligned total knee arthroplasty? limb and joint line alignment after kinematically aligned total knee arthroplasty. Bone Joint J.

[40] Sinicrope BJ, Feher AW, Bhimani SJ, Smith LS, Harwin SF, Yakkanti MR (2019). Increased survivorship of cementless versus cemented TKA in the morbidly obese. A minimum 5-year follow-up. J Arthroplasty.

[41] Victor JM, Bassens D, Bellemans J, Gursu S, Dhollander AA, Verdonk PC (2014). Constitutional varus does not affect joint line orientation in the coronal plane. Clin Orthop Relat Res.

[42] Workgroup of the American Association of Hip, Knee Surgeons Evidence Based Committee (2013). Obesity and total joint arthroplasty: a literature based review. J Arthroplasty.

[43] Yazdi H, Choo KJ, Restrepo C, Hammad M, Sherman M, Parvizi J (2020). Short-term results of triathlon cementless versus cemented primary total knee arthroplasty. Knee.

[44] Zhou K, Yu H, Li J, Wang H, Zhou Z, Pei F (2018). No difference in implant survivorship and clinical outcomes between full-cementless and full-cemented fixation in primary total knee arthroplasty: a systematic review and meta-analysis. Int J Surg.

