# Correction to: Restricted kinematic alignment leads to uncompromised osseointegration of cementless total knee arthroplasty

**DOI:** 10.1007/s00167-021-06488-w

**Published:** 2021-02-22

**Authors:** Guillaume Laforest, Lazaros Kostretzis, Marc-Olivier Kiss, Pascal-André Vendittoli

**Affiliations:** 1 Surgery Department, Hôpital Maisonneuve-Rosemont, Université de Montréal, 5415 Boul l’Assomption, Montreal, QC H1T 2M4 Canada; 2 Clinique Orthopédique Duval, Laval, QC Canada; 3 Personalized Arthroplasty Society, Montreal, Canada

## Correction to: Knee Surgery, Sports Traumatology, Arthroscopy https://doi.org/10.1007/s00167-020-06427-1

Authors would like to correct the error in in their original publication of the article.

Figure 3b and 3c have been switched. The image 3c should be 3b and 3b should be 3c. Correct version of Fig. 3 updated here.

The original article has been corrected.

**Fig. 3:**
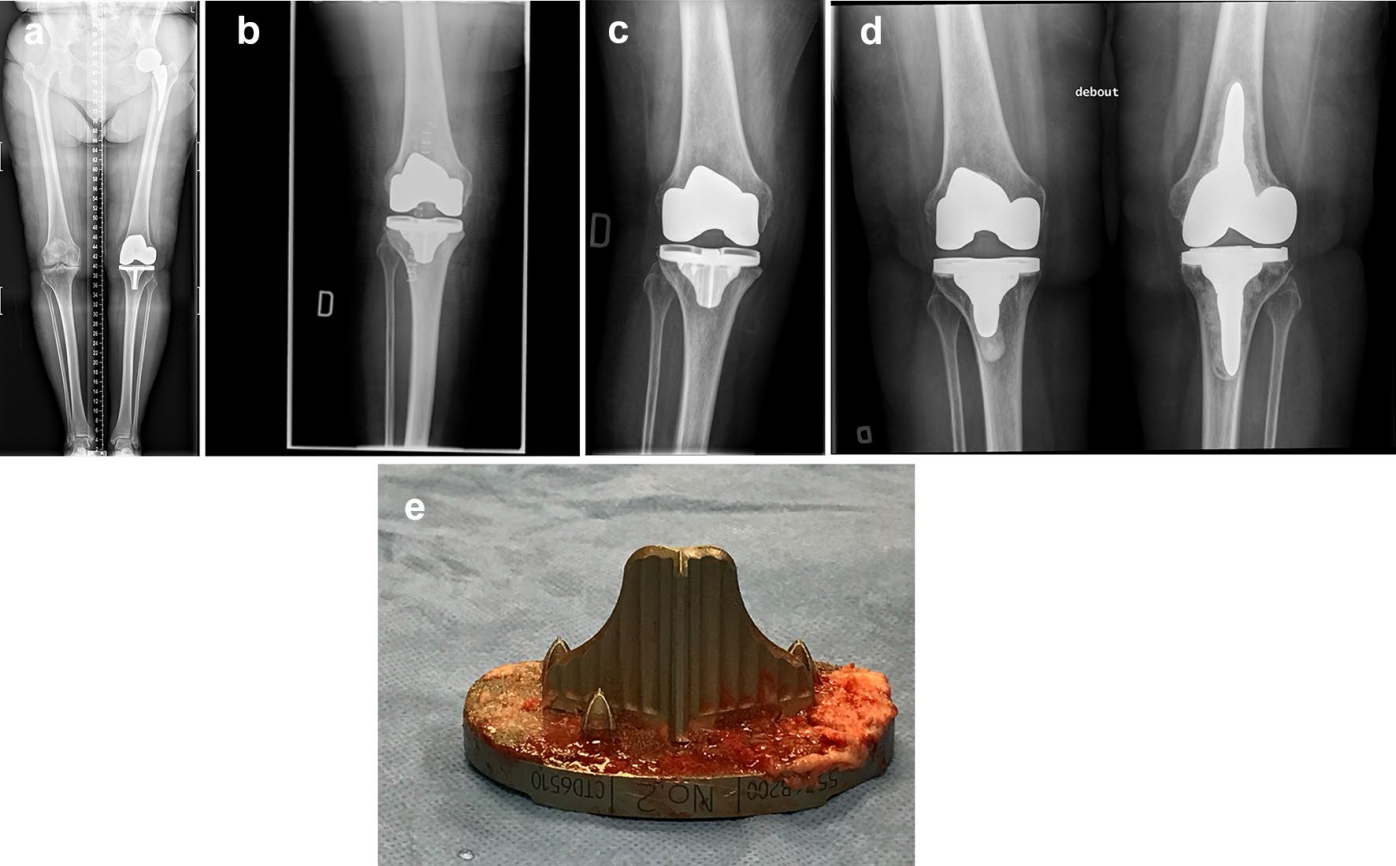
**a** Pre-op long leg AP view radiograph of a 76-year-old female with severe medial OA where LDFA is measured at 87.5° (valgus) and MPTA at 88.1° (varus), leading to an arithmetic HKA of 0.6°. **b** Immediate post-op AP radiograph showing uncemented TKA implants in acceptable orientation: 88.0° LDFA, 0° MPTA and arithmetic HKA of 2.0°. **c** Patient sustained a fall in stairs 4 weeks after surgery. Sudden and persisting pain and swelling were present. This is an AP view radiograph, 8 weeks post-op, showing a 5° valgus shift of the tibial implant (MPTA changed from 90° to 95° (LDFA was maintained at 88.0°). **d** Patient being unsatisfied with her lower limb alignment (HKA: 7° valgus) and having medial knee pain (medial collateral ligament over tensioned), she requested a TKA revision surgery. During revision procedure, the femoral implant was well fixed and considered well aligned. Tibial implant was revised alone, changing its orientation and using a cemented version. AP view radiograph post revision showing tibial implant’s MPTA at 88.0°, combined leading to an arithmetic HKA of 0° when combined with the femoral implant LDFA of 88.0°. **e** Removal of the well-fixed uncemented tibial implant was demanding, especially to break the osseous bonding behind the keel. Here is a photograph of the removed uncemented tibial implant where cancellous bone attachment is observed on the whole porous surface

